# Areas of professional life and job satisfaction of nurses

**DOI:** 10.3389/fpubh.2024.1370052

**Published:** 2024-04-04

**Authors:** Katarzyna Tomaszewska, Krystyna Kowalczuk, Bożena Majchrowicz, Alicja Kłos, Krzysztof Kalita

**Affiliations:** ^1^Department of Health Protection, Institute of Health Protection, The Bronislaw Markiewicz State Higher School of Technology and Economics, Jaroslaw, Poland; ^2^Department of Integrated Medical Care, Medical University of Bialystok, Bialystok, Poland; ^3^Department of Nursing, Institute of Health Protection, State Academy of Applied Sciences, Przemysl, Poland; ^4^Bureau of Statistical Research and Analysis, Rzeszow, Poland

**Keywords:** areas of professional life, professional work, job satisfaction, nurse, nursing

## Abstract

**Introduction:**

Job satisfaction among nurses is closely related to work environment as well as organizational and professional commitment. Satisfaction is a concept derived from Latin, where “satis” means “enough,” as much as is needed to fully satisfy expectations, needs, aspirations, in such a way that there is no room for complaint. Job satisfaction, on the other hand, is formulated as a positive attitude of employees toward the duties of the job, the work environment and other employees. The aim of this paper was to demonstrate how the different areas of nurses’ professional life, i.e., workload, control, rewards, community, sense of justice and values, correlate with their perceived job satisfaction.

**Materials and methods:**

A cross-sectional study was conducted in a group of 509 nurses working in a public hospital in Poland. Data were collected using a survey questionnaire, which consisted of a section containing sociodemographic data and standardized instruments: The Minnesota Satisfaction Questionnaire (MSQ) and The Areas of Worklife Survey (AWS) developed by Maslach and Leiter. Correlations were made using Spearman’s rho coefficient. The calculations also used stepwise linear regression analysis after checking certain assumptions, including checking the assumption of normality of residuals and the Durbin-Watson Test.

**Results:**

The mean score for the 20 items of the MSQ questionnaire ranged from 3.05 to 3.43 on a 5-point Likert scale. Support from the interdisciplinary team, which concerned assessing the quality of the social environment in the workplace, cooperation and showing positive feelings received the highest rating among respondents (3.51 ± 0.76). The sense of fair treatment at work averaged 3.26 ± 0.58. The area of value conflict within the organization itself or between the employee’s values and those of the organization, respondents rated an average of 3.26 ± 0.65. The mean score for all areas of professional work in the surveyed group was 3.09 ± 0.45.

**Conclusion:**

As satisfaction in particular areas of work life increases, so does the level of satisfaction in such aspects of work as achievement and a sense of fairness. The higher the level of satisfaction in the area of control, the more the sense of satisfaction with independence increases. The higher the satisfaction of respondents in the areas of values, workload and control, the higher the level of satisfaction with working conditions occurs.

## 1 Introduction

Work is the basic form of human activity. It is a value, a purposeful activity, a source of earnings, but also an opportunity to realize one’s own goals, desires or personal dreams. It allows to satisfy biological, social, economic and psychological needs, including the needs for success, belonging, recognition, respect and self-fulfillment, and is also included in the system of social interactions that shape one and affect the quality of one’s life (1–3). Nursing staff is considered a key part of the health care system and is the most numerous group of health care workers. However, shortages and high turnover rates have become a problem affecting the nursing profession in many countries (4). The work environment of nurses is important to the quality of patient care and is receiving increasing attention in research (5, 6).

Psychosocial burdens in the work environment have been analyzed for many years. There is no uniform definition that precisely defines the term psychosocial burden and classifies them. However, there is a common denominator that defines psychosocial burdens (stressors) as potential sources of stress that usually lead to psychological, physical, social harm. The most adequate definition of psychosocial burdens is that proposed by the WHO: “*...as a type of interaction between work content, work organization, management systems, environmental conditions and the competencies, needs and individual characteristics of the worker*” (7). Psychophysical load is a set of psychophysical demands to which an employee is subjected during the working day and is determined by the amount and type of information that a person has to deal with at work. Those that excessively affect employees cause various types of symptoms, including changes in personality such as sadness, anxiety, anger or devaluation, behavioral changes with deterioration in the quality of work, ineffective work performance and interpersonal conflicts (8, 9). The workload can also affect the quality of nursing care, through the significant strain of emotional demands, shift work and staff shortages (10, 11). This is one of the major concerns of nursing managers (9, 12–15).

The psychosocial work environment includes those factors that affect individuals and influence the health of employees, including both individual factors and the social work environment (16). They include the demands of the job: work organization, including influence, freedom, meaningful work and opportunities for growth; interpersonal relationships, such as leadership and co-workers, sense of community, role clarity, feedback and support; and individual health and personal factors, including coping ability and family support (17, 18).

Work environment is a well-known predictor of nurses’ job satisfaction as an external factor, while personal initiative may play a role as an intrapersonal (internal) trait (19, 20). Several factors contribute to the negative work environment. Traditionally, these include low wages and working conditions and a lack of respect for nurses, which has led to high turnover and an increase in the number of nurses leaving the profession. Other factors include lack of support, lack of staff and increased workload (21). The work environment also relates to so-called organizational characteristics that either facilitate or limit nursing practice. Lack of time, inadequacy of staff, and lack of team dynamics are potential factors that affect job satisfaction (22).

Job satisfaction can be defined in many ways. The earliest definition of job satisfaction was proposed by Hoppock (23) and reads as follows: “*the combination of psychological, physiological and environmental circumstances that cause a person to say: I am satisfied with my job.*” Wilson, on the other hand, defined job satisfaction as “*the degree to which needs are currently being met at work.*” Nelson and Quick defined it as “*a pleasant or positive emotional state resulting from an appraisal of one’s job or work experience*” (24). Job satisfaction is considered a global issue, but it is also necessary to improve the quality of care provided and cultivate an appropriate work environment in healthcare organizations, and a lack of job satisfaction among nurses can affect their practice, which in turn can directly or indirectly affect patient satisfaction (25, 26). A high level of job satisfaction can not only help medical personnel increase their self-confidence and professional identity, eliminate rationing of care, actively improve relationships with co-workers, but also effectively reduce the psychological stress that work brings (27, 28).

According to Brayer and Marcinowicz, the source of nurses’ greatest satisfaction with their work is contact with patients and their families, indicating the need for belonging in this professional group. Participation in the treatment process and the gratitude of patients and their families are other determinants of satisfaction, and reflect satisfaction of the need for respect and recognition. The sense of fulfillment, professional development and challenges at work are part of the need for self-realization, which is the highest level of human aspiration. The organization and conditions at a nurse’s workplace, relating to the satisfaction of the need for security, were the third most frequently cited reason for lack of job satisfaction after salary and lack of respect and recognition (29). Job satisfaction has been found to be related to the beliefs and emotions that individuals have about their jobs (26). The social prestige of the profession is also important, which, in addition to the desire to help others, may be a motive for choosing it (30).

The situation of nursing in Poland is difficult. It consists, in addition to those mentioned, of a low position among other medical professions, continuous improvement of qualifications and ethical requirements (31). In addition, the paternalistic model of patient care that functioned in health care for many years, where the nurse was perceived as subordinate to the doctor further influenced the low social status of this professional group. Research shows that improving nurses’ job satisfaction should be a key goal in the face of the challenges of achieving and maintaining quality standards, ensuring patient satisfaction and staff retention (32).

The prestige and importance of the professional group of nurses for achieving professional job satisfaction is extremely important. Success is determined by communication skills, group relations, cooperation skills and the degree of identification of nurses with the professional group (33). Therefore, it is crucial to examine how specific areas of nurses’ professional life, such as workload, control, rewards, community, a sense of justice and values, correlate with their perceived job satisfaction. Creating a friendly work environment in these areas may positively impact the level of satisfaction among nursing staff. Based on the main objective, the following research questions were formulated:

1. What is the level of job satisfaction of hospital nursing staff?
2. How do respondents rate the various areas of work life in their work environment?
3. What is the relationship between the various areas of nurses’ work life: workload, control, rewards, community and sense of justice and job satisfaction?

## 2 Materials and methods

### 2.1 Research design

In the present study, after obtaining approval from the Bioethics Committee, a survey was conducted among nurses employed in a state hospital in Poland who were actively working during the survey period. The survey was conducted between September and October 2022. The survey questionnaires were left in the nursing rooms with a request to fill them out, then they were collected by the authors of the study.

### 2.2 Research tools

The research tool was a survey questionnaire containing questions on sociodemographic data and standardized questionnaires: *The Minnesota Satisfaction Questionnaire* (MSQ) and *The Areas of Worklife Survey* (AWS) developed by Maslach and Leiter.

#### 2.2.1 The Minnesota Satisfaction Questionnaire

The Minnesota Satisfaction Questionnaire (MSQ) by Weiss et al. (34) is used to assess levels of job satisfaction and dissatisfaction. It assesses 20 job characteristics, such as achievement, independence, recognition, working conditions, among others. This abbreviated version of the MSQ questionnaire assessed intrinsic (i.e., what people think about their job duties) and extrinsic job satisfaction (i.e., what people think about aspects of work conditions that are external to the job itself). External, internal and overall job satisfaction scores are calculated by summing the corresponding subscale item scores. A five-point Likert scale is used in assessing satisfaction with the MSQ. The Cronbach’s alpha coefficient for the variables making up the MSQ in Polish was 0.858, which is a satisfactory level (35).

#### 2.2.2 The Areas of Worklife Survey

The Areas of Worklife Survey (AWS) is a tool constructed at the Canadian Centre for Organizational Research & Development at the University of Arcadia by Christina Maslach and Michael Leiter, who specialize in studying the phenomenon of burnout (36). Adaptation of the Polish version was carried out by Terlak and Izwantowska. All questions of the Polish version obtained a statistically significant correlation up to 0.05. As a measure of internal consistency of the test, Cronbach’s alpha coefficient of 0.83 was calculated. The questionnaire is designed for subjective assessment of the work environment by employees and covers issues such as workload, perceived support from the organization, support from other people at work, compatibility of the value system of the employee and the organization, degree of job satisfaction before salary regulation, degree of job satisfaction after salary regulation, degree of job satisfaction with one’s current nursing job, taking into account all aspects of work, including one’s own values, ideals and goals.

It allows the assessment of the employee’s functioning in the work environment and the incompatibility between the demands of the organization and the needs, aspirations and abilities of employees. The questionnaire consists of 29 statements grouped into six scales: workload, behavioral control, satisfaction with rewards, support from co-workers, sense of fairness and value scale (37).

### 2.3 Participants

The study group consisted of 509 nurses employed in hospital wards. Inclusion criteria were work as a nurse and consent to participate in the study. The signed statements are stored by the authors of this paper. Six hundred paper survey questionnaires were distributed with a request for completion, 518 were returned and, after final verification, 509 correctly completed ones were subjected to statistical analysis, accounting for 82.1%. The exclusion criterion was lack of consent to participate in the study.

### 2.4 Ethical procedure

The participation of nurses in the study was voluntary and anonymous. The study was conducted in accordance with the ethical standards set forth in the Declaration of Helsinki (64th WmA General Assembly, Fortaleza, Brazil, October 2013) and in accordance with Polish legal regulations. The application was favorably approved by the Bioethics Committee of the State Academy of Applied Sciences in Przemyśl (KBPANS 11/2022).

### 2.5 Statistical analysis

Correlations between ordinal or quantitative variables (when the conditions for using parametric tests were not met) were made using Spearman’s rho coefficient, which indicates the intensity of the relationship and its direction – positive or negative. The calculations used stepwise linear regression analysis after checking certain assumptions beforehand, including checking the assumption of normality of residuals and the Durbin-Watson test. The analysis was performed using the IBM SPSS 26.0 package (IBM, New York City, NY, United States) with the Exact Tests module. All correlations and differences are statistically significant when *p* < 0.05.

## 3 Results

### 3.1 Characteristics of the study group

97.2% of female nurses and only 2.8% of male nurses participated in the survey, therefore this variable was not considered in the statistical analysis. The age and job seniority of the respondents varied significantly. Detailed data are presented in Table 1.

**Table 1:** Characteristics of the study group.

Variable		Frequency (*n* = 509)	
Gender	Female	495	97.2%
	Male	14	2.8%
Age (years)	<30	143	28.1%
	30–40	129	25.3%
	41–50	149	29.3%
	>50	88	17.3%
Job seniority (years)	<5	94	18.5%
	5–10	164	32.2%
	11–15	48	9.4%
	16–20	55	10.8%
	>20	148	29.1%

### 3.2 Results of the Minnesota Satisfaction Questionnaire

The mean score for the 20-item MSQ questionnaire ranged from 3.05 to 3.43 on a 5-point Likert scale. Respondents’ level of satisfaction with their achievements at work averaged 3.05 ± 0.81 and their level of independence averaged 3.38 ± 0.66. Independence was 3.38 ± 0.66, recognition received the highest mean of 3.43 ± 0.87, and working conditions 3.19 ± 0.83. Considering all aspects of work, respondents rated their level of satisfaction at a mean of 3.58 ± 0.80 and satisfaction with their current life at 3.83 ± 0.77. In all sub-scales, the minimum number of points to be obtained was 1 point, and the maximum was 5 points. It can be inferred that the level of job satisfaction among the respondents was above average. The results are shown in Table 2.

**Table 2:** Results of MSQ job satisfaction questionnaire (*n* = 509).

	Mean	Median	Standard deviation
MSQ Achievements (1–5)	3.05	3.00	0.81
MSQ Independence (1–5)	3.38	3.40	0.66
MSQ Recognition (1–5)	3.43	4.00	0.87
MSQ Work conditions (1–5)	3.19	3.00	0.83
Considering all aspects of your job, to what extent are you satisfied? (1–5)	3.58	4.00	0.80
Overall, how satisfied are you with your current life? (1–5)	3.83	4.00	0.77

### 3.3 Results of the Areas of Worklife Survey

The Areas of Worklife Survey questionnaire was used to assess specific areas of nurses’ work environment: workload, level of control, rewards, community, sense of justice and values. The workload score of the nurses surveyed was 2.67 ± 0.72, slightly above the mean value (min. 1, max. 5). It can be assumed that the respondents are not overwhelmed by excessive workload. The ability to make independent decisions, to make choices on the job, the respondents rated a mean of 3.42 ± 0.79. This was one of the higher rated areas of work life, which also proved that nurses have a significant sense of control in their work. Another aspect was receiving both material rewards, opportunities for advancement, and social rewards such as recognition and respect from co-workers, superiors and clients. The mean score was 3.15 ± 0.66, indicating that nurses’ work is increasingly appreciated.

Support from the interdisciplinary team, which concerned the assessment of the quality of the social environment in the workplace, cooperation and the display of positive feelings received the highest score among the respondents. The mean score was 3.51 ± 0.76. The employee’s feeling of being treated fairly at his job averaged 3.26 ± 0.58. The final area was whether there was a conflict of values within the organization itself or between the employee’s values and those professed by the organization. This aspect of their work was rated by respondents at a mean of 3.26 ± 0.65. The mean score for all areas of professional work in the surveyed group was 3.09 ± 0.45 (max. 5).

Respondents also commented on salary regulation, which was in 2022 for the professional group of nurses. The level of job satisfaction before salary regulation averaged 2.81 ± 1.03 and after regulation 3.27 ± 1.03, a noticeable increase. The degree of satisfaction with one’s current nursing job, taking into account all aspects of work, including one’s own values, ideals and goals according to the respondents, averaged 5.93 ± 1.91 (max. 10) i.e. mean. The results are presented in Table 3.

**Table 3:** Results of AWS questionnaire (*n =* 509).

	Mean	Median	Standard deviation
Workload (1–5)	2.67	2.67	0.72
Control (1–5)	3.42	3.33	0.79
Rewards (1–5)	3.15	3.00	0.66
Community (1–5)	3.51	3.60	0.76
Sense of justice (1–5)	2.82	2.83	0.58
Values (1–5)	3.26	3.20	0.65
Overall (1–5)	3.09	3.10	0.45
Level of job satisfaction before salary regulation	2.81	3.00	1.03
Level of job satisfaction after salary regulation	3.27	3.00	1.03
Degree of satisfaction with one’s current nursing job, considering all aspects of the job, including one’s own values, ideals and goals	5.93	6.00	1.91

The calculations used linear regression analysis using the stepwise method after checking certain assumptions beforehand, including checking the assumption of normality of the residuals. In the method described above, only those predictors whose values proved to be statistically significant are included in the model. The selected dependent variables were the four MSQ domains. The adjusted predictors in the stepwise linear regression method explain a total of about 30% of the variance in the dependent variable in each case. The lack of correlation of the residuals is evidenced by the results of the Durbin Watson statistic, while a good fit of each model to the data is indicated by the “p” values obtained during the analysis of variance (*p* < 0.001). In the first model, where the dependent variable is MSQ-achievement four predictors proved statistically significant. The standardized regression coefficients report that as satisfaction increases in areas of work life such as sense of justice, control, rewards and values, satisfaction in an aspect of work such as achievement increases, with sense of justice being the more pronounced predictor (β = 0.257, *p* < 0.001). In the second model, where the dependent variable is MSQ-Independence, the same predictors also proved to be statistically significant, but the control factor proved to be a particularly clear value (β = 0.373, *p* < 0.001) - as satisfaction with control increases, the sense of satisfaction with independence increases. In the third model, where the dependent variable is recognition, the significant predictors turned out to be the above-mentioned variables in addition to rewards. Again, the most pronounced relationship is between control and recognition (β = 0.325, *p* < 0.001). With the last model, where the dependent variable is MSQ-work conditions, predictors such as values, workload and control turned out to be statistically significant. The higher the satisfaction in terms of these variables, the higher the level of satisfaction with working conditions. A slightly more pronounced coefficient value applies to β = 0.294 (*p* < 0.001). The results are presented in Table 4.

**Table 4:** Stepwise linear regression analysis.

	Stepwise linear regression					
	Dependent variable	*R*	Adjusted *R*-squared	Durbin-Watson statistics	*F*	*p*
MODEL A	MSQ Achievements (1–5)	0.55	0.297	2.017	54.720	<0.001
	Predicates	*B*	Standard error	*β*	*t*	*p*
	Sense of justice (1–5)	0.359	0.061	0.257	5.884	<0.001
	Control (1–5)	0.187	0.045	0.181	4.173	<0.001
	Rewards (1–5)	0.196	0.052	0.160	3.759	<0.001
	Values (1–5)	0.176	0.057	0.141	3.068	0.002
MODEL B	MSQ Independence (1–5)	0.581	0.333	1.971	64.370	<0.001
	Predicates	*B*	Standard error	*β*	*t*	*p*
	Control (1–5)	0.312	0.035	0.373	8.822	<0.001
	Values (1–5)	0.183	0.045	0.180	4.041	<0.001
	Sense of justice (1–5)	0.110	0.048	0.097	2.278	0.023
	Rewards (1–5)	0.092	0.041	0.093	2.232	0.026
MODEL C	MSQ Recognition (1–5)	0.49	0.235	2.071	53.110	<0.001
	Predicates	*B*	Standard error	*β*	*t*	*p*
	Control (1–5)	0.359	0.049	0.325	7.335	<0.001
	Values (1–5)	0.233	0.063	0.174	3.685	<0.001
	Sense of justice (1–5)	0.158	0.066	0.106	2.393	0.017
MODEL D	MSQ Work conditions (1–5)	0.559	0.308	2.035	76.487	<0.001
	Predicates	*B*	Standard error	*β*	*t*	*p*
	Values (1–5)	0.375	0.054	0.294	6.966	<0.001
	Workload (1–5)	0.301	0.044	0.262	6.904	<0.001
	Control (1–5)	0.225	0.044	0.214	5.107	<0.001

*F*, mean of the within-group variance. *R*, coefficient, measure of the quality of the model fit. *B*, value of the regression coefficient.

Age and length of service do not correlate statistically significantly with workload and degree of job satisfaction in the various aspects studied. Only four correlations were found to be statistically significant. Workload and working conditions (MSQ) correlate statistically significantly with seniority, while job satisfaction before wage regulation and achievement (MSQ) correlate statistically significantly with age.

The non-parametric Spearman’s rho was used in the calculations because variables such as age and length of service were of an ordinal and categorized nature in the survey. Age and length of service do not correlate statistically significantly with workload and degree of job satisfaction in the various aspects studied. Only four correlations were found to be statistically significant. Workload and working conditions (MSQ) correlate statistically significantly with seniority, while job satisfaction before wage regulation and achievement (MSQ) correlate statistically significantly with age. The results are presented in Table 5.

**Table 5:** Correlations between age, job seniority, results of the Areas of Worklife Survey (AWS), and the Minnesota Satisfaction Questionnaire (MSQ).

			Age	Job seniority
Spearman’s rho	Workload (1–5)	Correlation coefficient	−0.033	−0.088^*^
		Significance (two-tailed)	0.459	0.048
		Frequency (*n*)	509	509
	Control (1–5)	Correlation coefficient	0.024	0.004
		Significance (two-tailed)	0.591	0.933
		Frequency (*n*)	509	509
	Rewards (1–5)	Correlation coefficient	0.012	0.000
		Significance (two-tailed)	0.785	0.995
		Frequency (*n*)	509	509
	Community (1–5)	Correlation coefficient	−0.002	−0.032
		Significance (two-tailed)	0.961	0.470
		Frequency (*n*)	509	509
	Sense of justice (1–5)	Correlation coefficient	0.006	−0.015
		Significance (two-tailed)	0.894	0.732
		Frequency (*n*)	509	509
	Values (1–5)	Correlation coefficient	0.076	0.048
		Significance (two-tailed)	0.085	0.282
		Frequency (*n*)	509	509
	Total (1–5)	Correlation coefficient	0.026	−0.023
		Significance (two-tailed)	0.556	0.605
		Frequency (*n*)	509	509
	Summary: job satisfaction levels before salary regulation	Correlation coefficient	0.104^*^	0.053
		Significance (two-tailed)	0.019	0.233
		Frequency (*n*)	509	509
	Summary: level of job satisfaction after salary regulation	Correlation coefficient	0.032	0.020
		Significance (two-tailed)	0.472	0.645
		Frequency (*n*)	509	509
	MSQ achievements (1–5)	Correlation coefficient	0.106^*^	0.079
		Significance (two-tailed)	0.017	0.076
		Frequency (*n*)	509	509
	MSQ independence (1–5)	Correlation coefficient	0.031	0.030
		Significance (two-tailed)	0.489	0.502
		Frequency (*n*)	509	509
	MSQ Recognition (1–5)	Correlation coefficient	0.036	0.006
		Significance (two-tailed)	0.422	0.893
		Frequency (*n*)	509	509
	MSQ Work conditions (1–5)	Correlation coefficient	0.076	0.088^*^
		Significance (two-tailed)	0.089	0.048
		Frequency (*n*)	509	509
	Considering all aspects of your job, to what extent are you satisfied? (1–5)	Correlation coefficient	0.059	0.055
		Significance (two-tailed)	0.184	0.213
		Frequency (*n*)	509	509
	Overall, how satisfied are you with your current life? (1–5)	Correlation coefficient	0.048	0.049
		Significance (two-tailed)	0.281	0.271
		Frequency (*n*)	509	509

* Correlation significant at the 0.05 level (two-tailed).

## 4 Discussion

The aim of this paper was to research how the various areas of nurses’ work life: workload, control, rewards, community, sense of justice and values correlate with their perceived job satisfaction.

The mean score for the 20 items on the 5-point Likert scale of the MSQ job satisfaction questionnaire that was obtained in the survey ranged from 3.05 to 3.43. Respondents rated the lowest level of their own satisfaction with their achievements at work averaged 3.05 ± 0.81 and the highest was appreciation, which scored a mean of 3.43 ± 0.87. Perhaps this was due to the structure of the work - the only current career advancement is a management position, of which there is one per ward. There is no division between levels of practice and higher education and specialization can be completed by any nurse. Kovner et al. in their study showed that factors related to the work environment were significantly related to job satisfaction. More than 40.0% of the variation in satisfaction was explained by various attitudes at work: supervisor support, workgroup cohesiveness, work diversity, autonomy, organizational constraints, promotion opportunities, work and family conflict and fairness (38), which has been confirmed by other studies (28, 39, 40). In our study, the mean score of all areas of work life in the surveyed group was 3.09 ± 0.45 (max. 5) and the workload score of the surveyed nurses was 2.67 ± 0.72, which was slightly above the mean value, meaning that it was at a moderate level. Philips obtained different results in his study, showing that more than half of the respondents reported high workload and intention to leave their current position. Moderate to strong intercorrelations were found between perceptions of workload and intention to leave the nursing profession (41).

Other authors have shown that job satisfaction of nurses employed in hospitals is closely related to work environment, structural empowerment, organizational commitment, professional commitment, job stress, and patient satisfaction (20, 42–44). In the author’s study, workload in each field correlates statistically significantly with all aspects of job satisfaction. All correlations are positive, so it can be concluded that higher levels of satisfaction in areas of work life are associated with higher levels of satisfaction in every aspect of MSQ work. Other researchers also consider the work environment and aspects of work when assessing job satisfaction (19, 45). The influence of environmental factors on job satisfaction is important in nurses’ work. According to Albashayreh et al. increasing nurses’ participation in the affairs of the organization and providing adequate resources are the main ways to create a healthy work environment, which is a successful, cost-effective strategy for job satisfaction and thus retention of nurses (46).

Respondents also commented on the salary regulation, which was in 2022 for the professional group of nurses. The level of job satisfaction before wage regulation averaged 2.81 ± 1.03 and after regulation 3.27 ± 1.03, a noticeable increase. According to the authors, the current salary of nurses in Poland is satisfactory. Putra et al. proved that job satisfaction positively correlated with nurses’ work and four dimensions of job satisfaction, namely supervision, contingent rewards, coworkers, nature of work and communication dimensions, were positively correlated with nurses’ work activities (47). Other studies have shown that the most commonly identified predictors of job satisfaction were salary, organizational leadership and training opportunities, but the overall prevalence of job satisfaction among nurses was found to be low compared to global data (48, 49). A study by Gurkova et al. found weak to moderate positive correlations between nurses’ work environment and satisfaction with their nursing role, satisfaction with their current job, and satisfaction with teamwork. Nurses who considered leaving their current job or position rated their work environment significantly worse than nurses who did not intend to leave their jobs (50). Good relationships with co-workers in the Gawęda et al. study were the main source of satisfaction for respondents as were job security and a good work atmosphere. Most of the respondents were of the opinion that the work they were doing was rather satisfying (51). The authors of a cross-sectional study conducted among nurses in Poland and Norway came to important conclusions. It was shown that higher satisfaction with all the examined aspects of job satisfaction, except for satisfaction with salary, was characterized by nurses working in Norway. They showed higher overall satisfaction with life and work. Low and very low life satisfaction was declared by 75% of Polish nurses and only 16% from the Norwegian group, while very high life satisfaction was declared by almost one-fifth of nurses from Norway and only 1% from Poland (52). In the Bello et al. study, respondents expressed above-average satisfaction with only two aspects of their job, namely salary and creativity. Satisfaction was lowest for aspects of recognition, relationships and responsibility (53). In Walkowiak and Staszewski’s study, the most important factors that adversely affect nurses’ job satisfaction in the study group were salary, working conditions and hospital policies and practices. Factors positively affecting job satisfaction were job security, serving others and being active at work (54). The results of studies by other authors indicate that nurses’ job satisfaction is significantly lower when they work overtime and nurses with fewer absences are more satisfied with their work Nurses with longer tenure are more satisfied than those with shorter tenure, and nurses’ job satisfaction positively correlates with their intention to stay on the job which is a strong predictor of nursing turnover (55).

In our own study, the degree of satisfaction with one’s current nursing job, taking into account all aspects of work, including one’s own values, ideals and goals in the opinion of the respondents was at the mean of 5.93 ± 1.91 (max. 10). According to the authors, individual perceptions of the profession, empathy and a sense of professional identity, are important in this regard. A similar important conclusion was reached by Zdun et al. According to the authors, satisfaction with work builds self-esteem and knocks down the needs for self-realization. It is important that a satisfied employee shows more commitment, low absenteeism and his attitude to work and employer is more honest. Behavioral nurses surveyed were more satisfied with the opportunity for self-realization, pay and use of working time, and the ability to influence the course of work. Procedural nurses were most satisfied with freedom of action and subordination due to the nature of the work (56). Xu and Fan found that the impact of the work environment and the often limited level of patient trust in nurses influences the development of negative emotions in the nurse–patient relationship and significantly reduces job satisfaction (57), which has been confirmed by other studies (58, 59). In addition, the employment conditions of nursing staff can also affect their job satisfaction and emotional well-being. Safe working conditions can improve their job satisfaction and sense of commitment. Job satisfaction can be reduced by factors such as a lack of a sense of safe and healthy working conditions, failure to meet employees’ workload needs and expectations, and lack of independence (60).

To sum up, increasing the level of job satisfaction among nurses is a very important aspect, as this can both improve the quality of care received by patients and ensure the retention of nursing staff. In a situation where human life and health are at risk (as the time of the pandemic showed us, among other things), nurses, without paying attention to wages and workload, carried out their professional tasks at the highest level. Indirect correlations related to the work environment, social recognition or salary play an important role in the individual assessment of job satisfaction in our professional group.

## 5 Conclusion

As respondents’ contentment increases in such areas of work life as sense of justice, control, rewards and values, the level of satisfaction increases as well. A sense of fairness in the work aspect of achievement and recognition was meaningful to respondents. The higher the satisfaction in the area of control, the higher the sense of satisfaction with independence increases. Working conditions (MSQ) correlated statistically significantly with sense of value, workload, and control. The higher the satisfaction on these variables, the higher the level of satisfaction with working conditions. Among the areas of professional work rated lowest by the nurses surveyed and which could be improved were workload and sense of justice.

The results of the study can guide managers of health care units in creating a friendly work environment that will positively affect the satisfaction level of nursing staff. Further multicenter longitudinal studies are needed to generalize the findings and gain better insight into the causal relationship between perceived workload and nurses’ job satisfaction in order to make recommendations for healthcare managers.

## 6 Limitations of the study

Limitations characteristic of cross-sectional studies apply. The research was conducted in a single clinical hospital at a specific point in time. Additionally, during the survey, there was a possibility of exchanging opinions among nurses, which could influence response patterns.

## Data availability statement

The raw data supporting the conclusions of this article will be made available by the authors, without undue reservation.

## Ethics statement

The studies involving humans were approved by Bioethics Committee of the State Academy of Applied Sciences in Przemyśl. The studies were conducted in accordance with the local legislation and institutional requirements. The participants provided their written informed consent to participate in this study.

## Author contributions

KT: Conceptualization, Data curation, Formal analysis, Investigation, Methodology, Project administration, Resources, Supervision, Validation, Visualization, Writing – original draft, Writing – review & editing. KKo: Conceptualization, Data curation, Formal analysis, Investigation, Methodology, Resources, Supervision, Validation, Writing – review & editing. BM: Conceptualization, Data curation, Formal analysis, Investigation, Resources, Validation, Writing – review & editing. AK: Formal analysis, Investigation, Methodology, Resources, Validation, Writing – review & editing. KKa: Data curation, Formal analysis, Methodology, Validation, Writing – review & editing.

## References

[1] Lisowska E. Professional determinants of job satisfaction among teachers. Forum Pedagogiczne. (2017) 7:227–44. doi: 10.21697/fp.2017.1.16

[2] Olkiewicz J Andruszkiewicz A. The relationship between types of behavior and experience in labor and socio-demographic variables in a group of neurological nurses. Piel Neurolog I Neuroch. (2012) 2:70–5.

[3] Gorbaniuk J Chuchra M. The importance of work in the lives of men and women. Roczniki Teologiczne. (2019) 66:105–22. doi: 10.18290/rt.2019.66.1-7

[4] Marƒá M Bartosiewicz A Burzyńska J Chmiel Z Januszewicz P. A nursing shortage-a prospect of global and local policies. Int Nurs Rev. (2019) 66:9–16. doi: 10.1111/inr.12473, PMID: 30039849

[5] Maassen SM van Oostveen C Vermeulen H Weggelaar AM. Defining a positive work environment for hospital healthcare professionals: a Delphi study. PLoS One. (2021) 16:e0247530. doi: 10.1371/journal.pone.0247530, PMID: 33630923 PMC7906333

[6] Lee SE Scott LD. Hospital Nurses' work environment characteristics and patient safety outcomes: a literature review. West J Nurs Res. (2018) 40:121–45. doi: 10.1177/0193945916666071, PMID: 27586440

[7] Kowalczuk K Krajewska K Rolka H Kondzior D Sarnacka E. Psychosocial working conditions of nurses. Hygeia. Public Health. (2015) 50:621–9.

[8] Guz E Haratym I Wjciuk A Orze ÇZ. Effect of psychophysical loads in work. Anesthesia Nurse. (2021) 6:33–46. doi: 10.19251/pwod/2021.4(3)

[9] Maghsoud F Rezaei M Asgarian FS Rassouli M. Workload and quality of nursing care: the mediating role of implicit rationing of nursing care, job satisfaction and emotional exhaustion by using structural equations modeling approach. BMC Nurs. (2022) 21:273. doi: 10.1186/s12912-022-01055-1, PMID: 36209155 PMC9548180

[10] Chang LY Yu HH Chao YC. The relationship between nursing workload, quality of care, and nursing payment in intensive care units. J Nurs Res. (2019) 27:1–9. doi: 10.1097/jnr.0000000000000265, PMID: 29613879 PMC6369868

[11] Broetje S Jenny GJ Bauer GF. The key job demands and resources of nursing staff: an integrative review of reviews. Front Psychol. (2020) 11:84. doi: 10.3389/fpsyg.2020.00084, PMID: 32082226 PMC7005600

[12] Diehl E Rieger S Letzel S Schablon A Nienhaus A Escobar Pinzon LC. The relationship between workload and burnout among nurses: the buffering role of personal, social and organisational resources. PLoS One. (2021) 16:e0245798. doi: 10.1371/journal.pone.0245798, PMID: 33481918 PMC7822247

[13] Parola V Coelho A Neves H Bernardes RA Sousa JP Catela N. Burnout and nursing care: a concept paper. Nurs Rep. (2022) 12:464–71. doi: 10.3390/nursrep12030044, PMID: 35894034 PMC9326636

[14] Kabunga A Okalo P. Prevalence and predictors of burnout among nurses during COVID-19: a cross-sectional study in hospitals in Central Uganda. BMJ Open. (2021) 11:e054284. doi: 10.1136/bmjopen-2021-054284, PMID: 34593507 PMC8487018

[15] Helfrich CD Simonetti JA Clinton WL Wood GB Taylor L Schectman G. The association of team-specific workload and staffing with odds of burnout among VA primary care team members. J Gen Intern Med. (2017) 32:760–6. doi: 10.1007/s11606-017-4011-4, PMID: 28233221 PMC5481228

[16] Tomaszewska K Majchrowicz B Delong M. Impact of SARS-CoV-2 pandemic on psychosocial burden and job satisfaction of long-term care nurses in Poland. Int J Environ Res Public Health. (2022) 19:3555. doi: 10.3390/ijerph19063555, PMID: 35329241 PMC8953701

[17] Tomaszewska K Majchrowicz B Snarska K Guzak B. Psychosocial burden and quality of life of surveyed nurses during the SARS-CoV-2 pandemic. Int J Environ Res Public Health. (2023) 20:994. doi: 10.3390/ijerph20020994, PMID: 36673750 PMC9859002

[18] Donley J. The impact of work environment on job satisfaction: pre-COVID research to inform the future. Nurse Lead. (2021) 19:585–9. doi: 10.1016/j.mnl.2021.08.009, PMID: 34512206 PMC8416300

[19] Kagan I Hendel T Savitsky B. Personal initiative and work environment as predictors of job satisfaction among nurses: cross-sectional study. BMC Nurs. (2021) 20:87. doi: 10.1186/s12912-021-00615-1, PMID: 34090435 PMC8180055

[20] Lu H Zhao Y While A. Job satisfaction among hospital nurses: a literature review. Int J Nurs Stud. (2019) 94:21–31. doi: 10.1016/j.ijnurstu.2019.01.011 30928718

[21] De Oliveira DR Griep RH Portela LF Rotenberg L. Intention to leave profession, psychosocial environment and self-rated health among registered nurses from large hospitals in Brazil: a cross-sectional study. BMC Health Serv Res. (2017) 17:21. doi: 10.1186/s12913-016-1949-6, PMID: 28068999 PMC5223488

[22] Zhu X Zheng J Liu K You L. Rationing of nursing care and its relationship with nurse staffing and patient outcomes: the mediation effect tested by structural equation modeling. Int J Environ Res Public Health. (2019) 16:1672. doi: 10.3390/ijerph16101672, PMID: 31091660 PMC6572194

[23] Hoppock R. Job Satisfaction, Harper and Row, New York, (1935).

[24] Al-Haroon HI Al-Qahtani MF. The demographic predictors of job satisfaction among the nurses of a major public hospital in KSA. J Taibah Univ Med Sci. (2019) 15:32–8. doi: 10.1016/j.jtumed.2019.11.003, PMID: 32110180 PMC7033398

[25] Salahat MF Al-Hamdan ZM. Quality of nursing work life, job satisfaction, and intent to leave among Jordanian nurses: a descriptive study. Heliyon. (2022) 8:e09838. doi: 10.1016/j.heliyon.2022.e09838, PMID: 35815152 PMC9260616

[26] Lorber M Skela SB. Job satisfaction of nurses and identifying factors of job satisfaction in Slovenian hospitals. Croat Med J. (2012) 53:263–70. doi: 10.3325/cmj.2012.53.263, PMID: 22661140 PMC3368291

[27] Halcomb E Smyth E McInnes S. Job satisfaction and career intentions of registered nurses in primary health care: an integrative review. BMC Fam Pract. (2018) 19:136. doi: 10.1186/s12875-018-0819-1, PMID: 30086722 PMC6081816

[28] Liu D Yang X Zhang C Zhang W Tang Q Xie Y. Impact of job satisfaction and social support on job performance among primary care providers in Northeast China: a cross-sectional study. Front Public Health. (2022) 10:884955. doi: 10.3389/fpubh.2022.884955, PMID: 35801248 PMC9253396

[29] Brayer A Marcinowicz L. Professional satisfaction of masters of nursing versus Abraham Maslow’s hierarchy of needs — analysis of answers to open-ended questions. Problemy Pielęgniarstwa. (2014) 22:12–9.

[30] Hellín Gil MF Ruiz Hernández JA Ibáñez-López FJ Seva Llor AM Roldán Valcárcel MD Mikla M. Relationship between job satisfaction and workload of nurses in adult inpatient units. Int J Environ Res Public Health. (2022) 19:11701. doi: 10.3390/ijerph191811701 36141970 PMC9517381

[31] Kunecka D Skowron Ł. The model of professional satisfaction of nursing staff in Poland - brief communication. Int J Occup Saf Ergon. (2019) 25:646–9. doi: 10.1080/10803548.2018.1480152, PMID: 29790434

[32] Specchia ML Cozzolino MR Carini E Di Pilla A Galletti C Ricciardi W. Leadership styles and Nurses' job satisfaction. Results of a systematic review. Int J Environ Res Public Health. (2021) 18. doi: 10.3390/ijerph18041552, PMID: 33562016 PMC7915070

[33] Kociuba-Adamczuk K. Satisfaction of nurses with professional work In: Turowski K, editor. Profilaktyka i edukacja zdrowotna. Lublin: NeuroCentrum (2017), 123–140.

[34] Weiss D. J. Dawis R. V. England G. W. Lofquist L. H. (1967). Manual for the Minnesota Satisfaction Questionnaire. Minnesota Studies in Vocational Rehabilitation, Minneapolis: University of Minnesota, Vol. 22, Industrial Relations Center.

[35] Ingram T Głód W. Wykorzystanie MSQ jako narzędzia badania satysfakcji z pracy w wybranej jednostce ochrony zdrowia. Nauki o zarządzaniu. (2014) 3, 31–43.

[36] Leiter MP Maslach C. Six areas of worklife: a model of the organizational context of burnout. J Health Hum Serv Adm. (1999) 21:472–89. PMID: 10621016

[37] Terlak J Izwantowska A. Adaptation of the Areas of Worklife Survey C. Maslach and M. Leitera. Studia Psychologica UKSW. (2009) 9:223–32.

[38] Kovner C Brewer C Wu YW Cheng Y Suzuki M. Factors associated with work satisfaction of registered nurses. J Nurs Scholarsh. (2006) 38:71–9. doi: 10.1111/j.1547-5069.2006.00080.x 16579327

[39] Curtis EA Glacken M. Job satisfaction among public health nurses: a national survey. J Nurs Manag. (2014) 22:653–63. doi: 10.1111/jonm.12026 25041804

[40] Leong K Fong P Kuok C Meng L. Cross-sectional association and influencing factors of job satisfaction and burnout among nurses in Macao. SAGE Open. (2022) 12:11048. doi: 10.1177/21582440221104811

[41] Phillips C. Relationships between workload perception, burnout, and intent to leave among medical-surgical nurses. Int J Evid Based Healthc. (2020) 18:265–73. doi: 10.1097/XEB.0000000000000220, PMID: 32141948

[42] Niskala J Kanste O Tomietto M Miettunen J Tuomikoski AM Kyngs H. Interventions to improve nurses' job satisfaction: a systematic review and meta-analysis. J Adv Nurs. (2020) 76:1498–08. doi: 10.1111/jan.14342, PMID: 32128864

[43] Elbejjani M Abed Al Ahad M Simon M Ausserhofer D Dumit N Abu-Saad Huijer H. Work environment-related factors and nurses' health outcomes: a cross-sectional study in Lebanese hospitals. BMC Nurs. (2020) 19:95. doi: 10.1186/s12912-020-00485-z, PMID: 33061841 PMC7545948

[44] Chung HC Chen YC Chang SC Hsu WL Hsieh TC. Nurses' well-being, health-promoting lifestyle and work environment satisfaction correlation: a psychometric study for development of nursing health and job satisfaction model and scale. Int J Environ Res Public Health. (2020) 17:3582. doi: 10.3390/ijerph17103582, PMID: 32443758 PMC7277543

[45] Gillet N Fouquereau E Coillot H Cougot B Moret L Dupont S. The effects of work factors on nurses' job satisfaction, quality of care and turnover intentions in oncology. J Adv Nurs. (2018) 74:1208–19. doi: 10.1111/jan.13524, PMID: 29350770

[46] Albashayreh A Al Sabei SD Al-Rawajfah OM Al-Awaisi H. Healthy work environments are critical for nurse job satisfaction: implications for Oman. Int Nurs Rev. (2019) 66:389–95. doi: 10.1111/inr.12529, PMID: 31206654

[47] Putra KR Andayani T Ningrum EH. Job satisfaction and caring behavior among nurses in a military hospital: a cross-sectional study. J Public Health Res. (2021) 10:2212. doi: 10.4081/jphr.2021.2212, PMID: 33855409 PMC8129760

[48] Habtamu A Animut A Luel D. Job satisfaction among Ethiopian nurses: a systematic review. Front Nurs. (2021) 8:75–82. doi: 10.2478/fon-2021-0009

[49] Al Yahyaei A Hewison A Efstathiou N Carrick-Sen D. Nurses' intention to stay in the work environment in acute healthcare: a systematic review. J Res Nurs. (2022) 27:374–97. doi: 10.1177/17449871221080731, PMID: 35832883 PMC9272506

[50] Gurkov E Mikov Z Labudkov M Chocholkov D. Nurses' work environment, job satisfaction, and intention to leave - a cross-sectional study in Czech hospitals cent. Eur J Nurs Midw. (2021) 12:495–04. doi: 10.15452/cejnm.2021.12.0019

[51] Gawƒôda A Gönieek A Serysko B. Job satisfaction in the opinion of surveyed nurses. Nurs Public Health. (2018) 8:269–76. doi: 10.17219/pzp/91608

[52] Pawlik J Schneider-Matyka D Jurczak A Szkup M Grochans E. Assessment of satisfaction of nurses working in Poland and Norway Hygeia. Public Health. (2017) 52:249–54.

[53] Bello S Adewole DA Afolabi RF. Work facets predicting overall job satisfaction among resident doctors in selected teaching hospitals in southern Nigeria: a Minnesota satisfaction questionnaire survey. J Occup Health Epidemiol. (2020) 9:52–60. doi: 10.29252/johe.9.1.52

[54] Walkowiak D Staszewski R. The job satisfaction of polish nurses as measured with the Minnesota satisfaction questionnaire. J Public Health Nurs Med Rescue. (2019) 4

[55] Bragadóttir H Kalisch BJ Flygenring BG Tryggvadóttir GB. The relationship of nursing teamwork and job satisfaction in hospitals. SAGE Open Nurs. (2023) 9:1175027. doi: 10.1177/23779608231175027, PMID: 37214231 PMC10192802

[56] Zdun G Kopański Z Brukwicka I Jastrjemska S. Satisfaction with work. J Clin Healthcare. (2016) 4:16–21.

[57] Xu YW Fan L. Emotional labor and job satisfaction among nurses: the mediating effect of nurse-patient relationship. Front Psychol. (2023) 14:1094358. doi: 10.3389/fpsyg.2023.1094358, PMID: 37342648 PMC10278545

[58] Gulsen M Ozmen D. The relationship between emotional labour and job satisfaction in nursing. Int Nurs Rev. (2020) 67:145–54. doi: 10.1111/inr.12559 31746014

[59] Soriano-Vázquez I Cajachagua Castro M Morales-García WC. Emotional intelligence as a predictor of job satisfaction: the mediating role of conflict management in nurses. Front Public Health. (2023) 11:1249020. doi: 10.3389/fpubh.2023.1249020, PMID: 38026285 PMC10667434

[60] Mazuecos FJ De-Juanas Oliva Á Rodríguez-Bravo AE Páez GJ. The social values of nursing staff and the perceived quality of their professional lives. Healthcare (Basel). (2023) 11:2720. doi: 10.3390/healthcare11202720, PMID: 37893794 PMC10606655

